# Pre-Clinical Cell-Based Therapy for Limbal Stem Cell Deficiency

**DOI:** 10.3390/jfb6030863

**Published:** 2015-08-28

**Authors:** Amer Sehic, Øygunn Aass Utheim, Kristoffer Ommundsen, Tor Paaske Utheim

**Affiliations:** 1 Department of Oral Biology, Faculty of Dentistry, University of Oslo, Sognsvannsveien 10, Oslo 0372, Norway; E-Mail: amer.sehic@odont.uio.no; 2 Department of Ophthalmology, Oslo University Hospital, Kirkeveien 166, Oslo 0407, Norway; E-Mail: outheim@gmail.com; 3 Department of Medical Biochemistry, Oslo University Hospital, Kirkeveien 166, Oslo 0407, Norway; E-Mail: kristoffer@ommundsen.com

**Keywords:** biomaterials, cornea, *ex vivo* cultivation, limbal stem cell deficiency, ocular surface disease, transplantation

## Abstract

The cornea is essential for normal vision by maintaining transparency for light transmission. Limbal stem cells, which reside in the corneal periphery, contribute to the homeostasis of the corneal epithelium. Any damage or disease affecting the function of these cells may result in limbal stem cell deficiency (LSCD). The condition may result in both severe pain and blindness. Transplantation of *ex vivo* cultured cells onto the cornea is most often an effective therapeutic strategy for LSCD. The use of *ex vivo* cultured limbal epithelial cells (LEC), oral mucosal epithelial cells, and conjunctival epithelial cells to treat LSCD has been explored in humans. The present review focuses on the current state of knowledge of the many other cell-based therapies of LSCD that have so far exclusively been explored in animal models as there is currently no consensus on the best cell type for treating LSCD. Major findings of all these studies with special emphasis on substrates for culture and transplantation are systematically presented and discussed. Among the many potential cell types that still have not been used clinically, we conclude that two easily accessible autologous sources, epidermal stem cells and hair follicle-derived stem cells, are particularly strong candidates for future clinical trials.

## 1. Cornea and Limbal Stem Cells

The cornea is the anterior, transparent, and avascular tissue with high refractive power that directs light bundles to the retina [1]. The highly specialized structure of the cornea is essential for normal vision. From anterior to posterior, the cornea is composed of five layers, *i.e.*, epithelium, Bowman’s membrane, stroma, Descemet’s membrane, and endothelium. The corneal epithelium is composed of a basal layer of column-shaped cells, a suprabasal layer of cuboid wing cells, and a superficial layer of flat squamous cells [2]. The thickness of the corneal epithelium in different species, e.g., human, mouse, and rabbit, is conspicuously perpetual, ranging from 45 to 50 μm [3,4,5]. The renewal of corneal epithelium differs between species and is renewed every 9–12 months in rabbits [6]. The corneal epithelium plays an essential role in maintaining the cornea’s avascularity and transparency [7]. 

The self-renewing properties of the corneal epithelium are an important requirement for corneal integrity and function [8]. This process is dependent on a small population of limbal stem cells that are situated in the basal region of the limbus [9,10]. Limbal stem cells are presented in the basal layer of the limbal epithelium and give rise to fast-dividing, transient amplifying cells [11]. Transient amplifying cells go through a restricted number of divisions before becoming terminally differentiated cells [12]. It has been hypothesized that corneal epithelial maintenance can be defined by the equation *X* + *Y* = *Z*, in which *X* refers to proliferation of basal cells; *Y* is the centripetal movement of peripheral cells; and *Z* is the epithelial cell loss from the corneal surface [13].

## 2. Limbal Stem Cell Deficiency

Any process or disease that results in dysfunction or loss of the limbal epithelial cells (LEC) may result in limbal stem cell deficiency (LSCD) [7]. In LSCD, the conjunctival epithelium migrates across the limbus, resulting in loss of corneal clarity and visual impairment. The condition is painful and potentially blinding [14]. Normal and well-functioning LEC act as an important barrier, preventing invasion of the cornea by conjunctival tissue. Limbal stem cell deficiency typically worsens over time since chronic inflammation not only results in the death of LEC, but also negatively affects the remaining stem cells and their function [14].

The prevalence and incidence of LSCD worldwide are not known. In India, the prevalence is estimated to be approximately 1.5 million [15], and the incidence in North America is estimated to be “thousands” [16]. The etiology of many cases of LSCD is known; however, idiopathic cases also exist [17,18]. Acquired causes of LSCD include thermal and chemical burns of the ocular surface, contact lens wear, ultraviolet radiation, extensive cryotherapy, or surgery to the limbus [7]. There are also numerous hereditary causes of LSCD, including aniridia, where the anterior segment of the eye including the limbus is imperfectly developed. Furthermore, autoimmune diseases involving the ocular surface, e.g., Stevens-Johnson syndrome and ocular cicatricial pemphigoid, are examples of nonhereditary causes of LSCD.

Limbal stem cell deficiency is classified as either partial or total, depending on the extent of the disorder. Conjunctivalization is pathognomonic for LSCD. Other signs are persistent epithelial defects, superficial and deep corneal vascularization, and fibrovascular pannus. Limbal stem cell deficiency in patients with significantly dry eyes results in a partial or total keratinized epithelium [19]. The diagnosis can be corroborated by detection of conjunctival cells on the corneal surface by cytological analysis [20] or *in vivo* confocal microscopy [21], but is seldom performed as the diagnosis is often obvious.

## 3. Treatment Approaches for Limbal Stem Cell Deficiency

The core of conservative treatment for LSCD lies in the improvement of epithelial healing. A range of clinical procedures, with distinctive benefits and limitations, are currently available for treating LSCD. However, variations in both the severity and causes of LSCD explain why the application of one treatment approach will not be adequate for all. A great variety of cell-based therapeutic strategies have been suggested for LSCD over the past 10 years. In cases of partial LSCD, amniotic membrane (AM) can be applied to the affected eye and aids in repopulating the ocular surface with corneal epithelium [22]. With increased understanding of the origin of the stem cells in the limbus [10], the transplantation of limbal grafts was introduced in 1989 [23], a promising treatment strategy for restoring the ocular surface following LSCD. This procedure, however, carried a risk of inducing LSCD in the healthy eye due to the need of large limbal biopsy, making the therapy impossible in cases of bilateral LSCD.

In 1997, a groundbreaking therapeutic strategy involving *ex vivo* expansion of LEC was introduced [24]. The principle of this method is to culture LEC harvested from the patient, a living relative, or a cadaver on a substrate in the laboratory and then transfer the cultured tissue onto the eyes of patients suffering from LSCD. This therapy has gained popularity in ophthalmology as it increases cell numbers before transplantation without the need for a large limbal biopsy. It is suggested that the mechanism underlying the improvement in the ocular surface after LEC allograft transplantation is due to the stimulation of a small number of residual dormant host cells, rather than transplanted cells, permanently replacing the ocular surface [25]. Another possibility is that the transplanted graft somehow attends to stimulate progenitor cells in the blood stream to repopulate the ocular surface [25].

Recently, the use of induced pluripotent stem cells (iPSCs) has attracted great attention [26,27]. Following culture for two weeks on an amniotic membrane, limbal iPSCs developed substantially higher expression of several putative limbal stem cell markers, including ABCG2 and ΔNp63α, than did fibroblast iPSCs [27]. The successful generation of iPSCs from human primary LEC, and subsequent re-differentiation back to the limbal corneal epithelium, has been demonstrated *in vitro* [27]. However, IPSCs have so far not been used in clinical studies or experimental animals for ocular surface reconstruction, despite the great promise this treatment holds.

Since 1997, several research groups have shown favorable effects of *ex vivo* cultured cell therapy for LSCD in both clinical studies and experimental animals. There is currently a strong trend toward applying autologous sources as there is no risk for immunological reactions and, therefore, no requirement for immunosuppressive therapy with all known side effects [28]. Since 2003, several non-limbal cells have been successfully used to reconstruct the corneal epithelium in bilateral LSCD, in which limbal tissue is not recommended for harvest. Among non-limbal cell types, oral mucosal epithelial cells and conjuctival epithelial cells are the only laboratory cultured cell sources that have been explored in humans. Oral mucosal epithelial cells were the first non-limbal cell type to be identified as a potential source for LSCD. So far, 242 patients have been reported to be treated with a success rate of 72% [29]. Since 2009, conjunctival epithelial cells have also been used with the purpose of reverting LSCD in clinical trials, but the number of patients treated is small [30]. Since 2010, there have been two clinical studies including 17 eyes that have used nasal mucosal epithelial cells to treat LSCD with promising results [31,32]. In contrast to most of the other cell types that have been used for LSCD therapy, nasal mucosa was transplanted to the eyes without prior *ex vivo* cultivation, which substantially simplifies the procedure.

**Figure 1:**
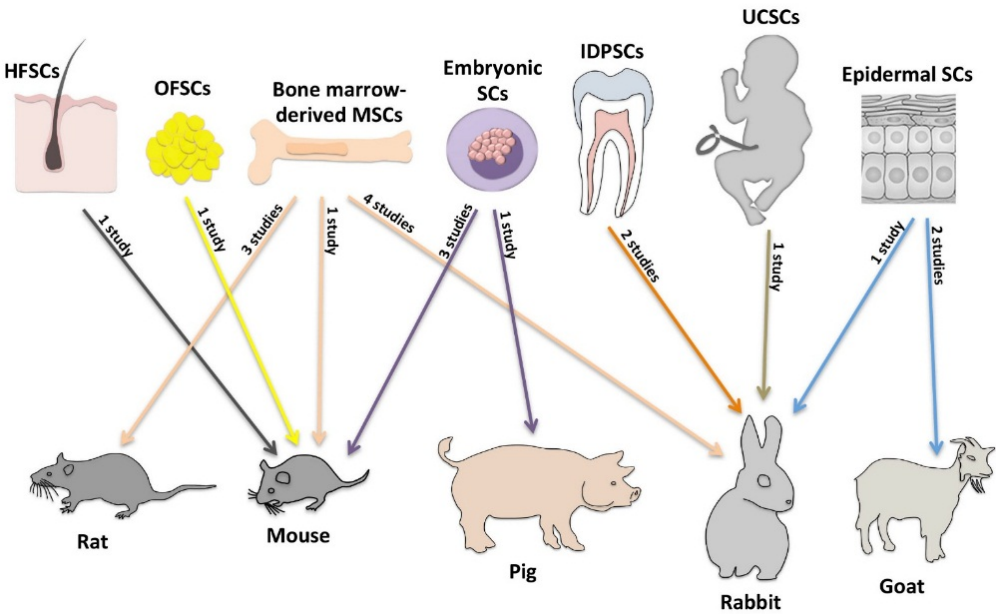
Overview of stem cell sources used in animal experiments. Arrows, including number of studies, indicate the connection between different stem cell sources and LSCD animal models that they have been transplanted to. HFSCs, hair follicle-derived stem cells; MSCs, mesenchymal stem cells; SCs, stem cells; IDPDSCs, immature dental pulp stem cells; OFSCs, orbital fat-derived stem cells; UCSCs, umbilical cord stem cells.

A number of other non-ocular cells have been investigated as alternative stem cell sources for treating LSCD; however, they have only been studied in animal experiments. As none of the cell types used in clinical trials have proved to be successful in more than about three of four cases [7,29], there has been a constant search for novel cell types that potentially could be more effective in reverting LSCD. The present review focuses on these cell types. The review was prepared by searching the National Library of Medicine database using the broad search term “limbal” in an attempt not to leave out any relevant publications. In total, the search resulted in 3634 studies, whereof 19 studies, published from 2004 to 2014, were related directly to the core topic of the present review. These studies include the following cultured cell types: (1) bone marrow-derived mesenchymal stem cells (Table 1) [33,34,35,36,37,38,39,40]; (2) embryonic stem cells (Table 2) [41,42,43,44]; (3) epidermal stem cells (Table 3) [45,46,47]; (4) hair follicle-derived stem cells (Table 4) [48]; (5) immature dental pulp stem cells (Table 4) [49,50]; (6) orbital fat-derived stem cells (Table 4) [51]; and (7) umbilical cord stem cells (Table 4) [52]. Various substrates and methods have been applied to culture and transplant these cell sources onto damaged corneas of mice, rats, rabbits, pigs, and goats (Figure 1, Table 5). In the present review, the ability of all these cell sources to treat LSCD is discussed.

**Table 1:** Reconstruction of ocular surface using cultured bone marrow-derived mesenchymal stem cells.

Author, Year, (Reference)	Cell Source	Methods	LSCD Model	Follow-up Time	Evaluation	Results
Ma *et al.* 2006 [35]	Bone Marrow-Derived MSCs; Human	Cultured on AM carrier; Transplanted (*n =* 16); Control groups: 1) transplanted with fibroblast cells on AM (*n =* 8) and 2) transplanted with only AM (*n =* 7)	Rats; Disc paper saturated with 1 N NaOH onto cornea	4 weeks	Slit lamp evaluation; Histology; IH	Reconstruction in 100% (16/16) of animals; Cornea completely transparent in 56.3% (9/16) of animals; Neovascularization detected within 2 mm and over 2 mm in 37.5% (6/16) and 12.5% (2/16) of animals, respectively; No complications
Ye *et al.* 2006 [39]	Bone Marrow-Derived MSCs; Rabbit	Cultured in α-MEM; IV injection; Four groups: 1) normal BM function, without MSCs injection (*n =* 15), 2) normal BM function, with MSCs injection (*n =* 15), 3) BM suppressed by CP, without MSCs injection (*n =* 15), 4) BM suppressed by CP, with MSCs injection (*n =* 15)	Rabbits; Filter paper saturated with 1 N NaOH onto cornea	1 month	Slit lamp evaluation; IH	Reconstruction in 100% (15/15) of animals in Group 2; Cornea more clear in group 2 compared with other groups; Neovascularization appeared on day 14 in Group 2; No complications
Gu *et al.* 2009 [33]	Bone Marrow-Derived MSCs; Rabbit	Cultured on fibrin carrier; Transplanted (*n =* 10); Control: eyes transplanted with only fibrin graft gel (*n =* 10)	Rabbits; Cornea treated with n-heptanol	4 weeks	Slit lamp evaluation; Histology; FC; IF	Reconstruction in 100% (10/10) of animals; Iris partially clear in 30% (3/10) and completely obscure in 70% (7/10) of animals; Neovascularization detected over 3 mm from the limbus in 80% (8/10) of animals; No complications
Omoto *et al.* 2009 [36]	Bone Marrow-Derived MSCs; Human	Cultured in α-MEM; Carrier-free sheets transplanted; Control: no transplantation; Number of animals not reported	Rabbits; Cornea treated with n-heptanol	4 weeks	Slit lamp evaluation; Histology; IH; RT-PCR	Reconstruction of corneal epithelium successful; Corneal clarity: no data; Neovascularization: no data; No complications
Jiang *et al.* 2010 [34]	Bone Marrow-Derived MSCs; Rat	Cultured on AM carrier; Three groups: 1) transplanted with only AM (*n =* 12); 2) MSCs on AM (*n =* 12); 3) MSCs induced by CSCs on AM (*n =* 12); Control: no transplantation (*n =* 12)	Rats; Filter paper saturated with 1 N NaOH onto cornea	10 weeks	Slit lamp evaluation; Histology; CLCM; SEM; FC; IF; IH	Reconstruction in 75% (9/12) of animals in group 3; Cornea completely transparent in 75% (9/12) of animals; Neovascularization limited within 2 mm of the limbus; No complications
Zajicova *et al.* 2010 [40]	Bone Marrow-Derived MSCs; Mouse	Cultured on nanofiber scaffold carrier; Co-transplantation of LSC and MSCs; Control: normal eyes; Number of animals not reported	Mice; Epithelial debridement with a needle	2 weeks	Slit lamp evaluation; CLCM; FC; RT-PCR	Significantly inhibited local inflammatory reactions and supported healing process; Corneal clarity: no data; Neovascularization: no data; No complications
Reinshagen *et al.* 2011 [37]	Bone Marrow-Derived MSCs; Rabbit	Cultured in DMEM; Three groups: 1) MSCs injected under transplanted AM (*n =* 6); 2) transplanted with only AM (*n =* 5); 3) transplanted with AM and autologous LEC (*n =* 4), Control: no transplantation (*n =* 6)	Rabbits; Cornea treated with n-heptanol	6 months	Slit lamp evaluation; Histology; IH	Reconstruction in 100% (6/6) of animals in Group 1; Improved corneal clarity; Neovascularization of the entire cornea in all animals; No complications
Rohaina *et al.* 2014 [38]	Bone Marrow-Derived MSCs; Human	Cultured on AM carrier; Transplanted (*n =* 4); Control groups: 1) transplanted with only AM (*n =* 5); 2) no transplantation (*n =* 6)	Rats; Disc paper saturated with 1 N NaOH onto cornea	8 weeks	Slit lamp evaluation; Histology; IH; OCT; RT-PCR	Reconstruction in 100% (4/4) of animals; Moderate corneal clarity; Minimal vascularization; No complications

AM, amniotic membrane; BM, bone marrow; CFE, colony-forming efficiency; CLCM, confocal laser corneal microscopy; CP, cyclophosphamide; CSCs, corneal stromal cells; FC, flow cytometry; IH, immunohistochemistry; IF, immunofluorescence; IV, intravenous; LEC, limbal epithelial cells; LSC, limbal stem cells; LSCD, limbal stem cell deficiency; MSCs, mesenchymal stem cells; OCT, optical coherence tomography; RT-PCR, reverse transcriptase polymerase chain reaction; SEM, scanning electron microscopy.

**Table 2:** Reconstruction of ocular surface using cultured embryonic stem cells.

Author, Year, (Reference)	Cell Source	Methods	LSCD Model	Follow-up Time	Evaluation	Results
Homma *et al.* 2004 [41]	Embryonic SCs; Mouse	Cultured on collagen IV-coated plates; Carrier-free sheets transplanted (*n =* 10); Control: no transplantation (*n =* 10)	Mice; Cornea treated with n-heptanol	24 h	FC; Histology; RT-PCR; WB	Reconstruction in 100% (10/10) of animals; Corneal clarity: no data; Neovascularization: no data; No complications
Ueno *et al.* 2007 [44]	Embryonic SCs; Mouse	Cultured on gelatin-coated plates; Transfected with Pax6; Carrier-free sheets transplanted (*n =* 5); Control groups: 1) normal eyes (*n =* 5); 2) no transplantation (*n =* 5)	Mice; Cornea treated with n-heptanol	24 h	Histology; IF; RT-PCR	Reconstruction in 100% (5/5) of animals 12 h after transplantation; Corneal clarity: no data; Neovascularization: no data; No complications
Kumagai *et al.* 2010 [42]	Embryonic SCs; Monkey	Cultured on collagen IV-coated plates; Carrier-free sheets transplanted (*n =* 10); Control groups: 1) normal eyes (*n =* 10); 2) no transplantation (*n =* 10)	Mice; Cornea treated with n-heptanol	6 h	CLCM; IF; RT-PCR	Transplanted cells adhered to the corneal stroma and formed multiple cell layers in 100% (10/10) of animals; Corneal clarity: no data; Neovascularization: no data; No complications
Notara *et al.* 2013 [43]	Embryonic SCs; Mouse	Cultured on collagen IV-coated plates; Carrier-free sheets transplanted; Control: no transplantation; Number of animals not reported	Pigs; Epithelial debridement with a blade	5 weeks	Histology; IH; RT-PCR; WB	Reconstruction after 1 week; Corneal clarity: no data; Neovascularization: no data; Mild immune reaction

CLCM, confocal laser corneal microscopy; FC, flow cytometry; IF, immunofluorescence; IH, immunohistochemistry; RT-PCR, reverse transcriptase polymerase chain reaction; SCs, stem cells; SEM, scanning electron microscopy; WB, western blotting.

**Table 3:** Reconstruction of ocular surface using cultured epidermal stem cells.

Author, Year, (Reference)	Cell Source	Methods	LSCD Model	Follow-up Time	Evaluation	Results
Yang *et al.* 2007 [47]	Epidermal SCs; Goat	Cultured on AM carrier; Transplanted (*n =* 7); Control groups: 1) transplantation with AM (*n =* 4); 2) no transplantation (*n =* 4)	Goats; Excision of the cornea and limbus	24 months	IH; SEM; TEM	Reconstruction in 100% (7/7) of animals; Two or three quadrants of clear cornea in 71.4% (5/7) of animals at follow-up time to 24 months; Minimal neovascularization; Perforation through the pupil during operation in one eye
Yang *et al.* 2008 [46]	Epidermal SCs; Goat	Cultured on AM carrier; Transplanted (*n =* 10); Control groups: 1) transplanted with only AM (*n =* 8); 2) no transplantation (*n =* 8)	Goats; Excision of the cornea and limbus; Burned with 1 N NaOH	30 months	Digital camera; Histology; IH	Reconstruction in 100% (10/10) of animals; Three or four quadrants of clear cornea in 80% (8/100) of animals at follow-up time to 30 months; Minimal neovascularization; No complications
Ouyang *et al.* 2014 [45]	Epidermal SCs; Human	Cultured on fibrin carrier; Transduction of Pax6 converted these cells into LSC-like cells; Transplanted and covered with AM (*n =* 5); Control: transplanted with only AM (*n =* 4)	Rabbits; Excision of the cornea and limbus	3 months	CLCM; IF; Microarrays; Quantitative PCR; RNA-sequencing; WB	Reconstruction in 100% (5/5) of animals; Transparent cornea in 100% (5/5) of animals for over 3 months; Minimal neovascularization; No complications

AM, amniotic membrane; CLCM, confocal laser corneal microscopy; IF, immunofluorescence; IH, immunohistochemistry; LEC, limbal epithelial cells; LSC, limbal stem cells; LSCD, limbal stem cell deficiency; PCR, polymerase chain reaction; SCs, stem cells; SEM, scanning electron microscopy; TEM, transmission electron microscopy; WB, western blotting.

**Table 4:** Reconstruction of ocular surface using cultured immature dental pulp stem cells, hair follicle-derived stem cells, umbilical cord stem cells, and orbital fat-derived stem cells.

Author, Year, (Reference)	Cell Source	Methods	LSCD Model	Follow-up Time	Evaluation	Results
Monteiro *et al.* 2009 [50]	IDPSCs; Human	Cultured on AM carrier; Transplanted (*n =* 5); Control: transplanted with only AM (*n =* 5)	Rabbits; Chemical burn of the cornea	3 months	Slit lamp evaluation; CLCM; IF; RT-PCR	Reconstruction in 100% (5/5) of animals; Gradual improvement in corneal transparency in 100% (5/5) of animals during follow-up time of 3 months; Neovascularization: no data; No complications
Gomes *et al.* 2010 [49]	IDPSCs; Human	Cultured on AM carrier; MCB (*n =* 5), SCB (*n =* 4); Transplanted and covered with AM; Control: transplanted with only AM (*n =* 6)	Rabbits; Filter paper saturated with 0.5 M NaOH for 25 s (MCB), and for 45 s (SCB)	3 months	Slit lamp evaluation; EM; Histology; IH	Reconstruction in 100% (5/5) of animals; Less organized and loose corneal epithelium in 75% (3/4) of SCB animals; Improved corneal clarity in 100% (5/5) of MCB animals; Superficial neovascularization in one animal No complications
Meyer-Blazejewska *et al.* 2011 [48]	HFSCs; Mouse	Cultured on fibrin carrier; Transplanted (*n =* 31); Control: no transplantation (*n =* 31)	Mice; Cornea and limbus removed	5 weeks	Slit lamp evaluation; Histology; IF	Reconstruction in 87.5% (7/8) of animals after two weeks Improved corneal clarity; Neovascularization in 12.5% (1/8) of animals; No complications
Reza *et al.* 2011 [52]	UCSCs; Human	Cultured on AM carrier; Three groups: 1) transplanted cell sheets on AM (*n =* 6); 2) transplanted with only AM; 3) no transplantation	Rabbits; Cornea and limbus removed	4 weeks	Slit lamp evaluation; Histology; IC; IH; RT-PCR	Reconstruction in 66.7% (4/6) of animals; Corneal clarity: no data; Severe neovascularization in one eye; Mild superficial inflammation in one other
Lin *et al.* 2013 [51]	OFSCs; Human	Cultured in MesenPro medium; Topical application of cells (*n =* 9), Intra-limbal injection of cells (*n =* 3); Control: Topical application of PBS (*n =* 6), Injection of PBS (*n =* 3), no treatment (*n =* 3)	Mice; Filter paper saturated with 0.5 N NaOH onto cornea	1 week	Digital camera; Histology; IH; IF; WB	Reconstruction of corneal epithelium after 1 week; Improved corneal clarity; No neovascularization; No complications

AM, amniotic membrane; CLCM, Confocal laser corneal microscopy; EM, electron microscopy; HFSCs, hair follicle-derived stem cells; IC, immunocytochemistry; IF, immunofluorescence; IH, immunohistochemistry; IDPSCs, immature dental pulp stem cells; LSC, limbal stem cells; LSCD, limbal stem cell deficiency; MCB, mild chemical burn; OFSCs, orbital fat-derived stem cells; PBS, phosphate buffered saline; RT-PCR, reverse transcriptase polymerase chain reaction; SC, stem cell; SCB, severe chemical burn; UCSCs, umbilical cord stem cells.

**Table 5:** Different culture and carrier biomaterials and methods used in cell-based therapies of LSCD, explored in animal models.

Methods	Materials	References
Transplantation	Carrier-free cell sheets	[36,41,42,43,44]
Transplantation	Amniotic membrane	[34,38,46,47,49,50,52,53]
Intravenous injection	–	[39]
Transplantation	Fibrin scaffold	[33,45,48]
Transplantation	Nanofiber scaffold	[40]
Injection under amniotic membrane	–	[37]
Topical application/Intra-limbal injection	–	[51]

## 4. Substrates for Corneal Reconstruction

To what extent biomechanical properties of the underlying substrate determine the success of *ex vivo* expansion of stem cells in treatment of LSCD is unknown. It is reasonable to assume that the optimal substrate will at least in some way resemble the limbal niche, in which limbal stem cells reside. The most common culture substrate for corneal reconstruction has so far been human AM. However, a number of alternative biological, biosynthetic, or synthetic substrates have been suggested as potential materials for ocular surface reconstruction (Table 6). The fundamental characteristics of an appropriate scaffold include cell attachment and cell proliferation both in culture and after transplantation, transparency, mechanical stability, and biocompatibility. In the studies on cell-based therapies for LSCD that have only been investigated in animal experiments, three substrates have so far been used: AM [34,38,46,47,49,50,52,53], nanofiber scaffold [40], and fibrin scaffold [33,45,48]. In addition, carrier-free methods [36,41,42,43,44], transplanting intact cell sheets without an underlying supportive membrane, injection of cells under transplanted AM [37], topical application of cells [51], intra-limbal injection of cells [51], and intravenous injection through an ear vein [39] have been applied (Table 5).

Amniotic membrane promotes cellular growth and exhibits anti-angiogenic and anti-inflammatory characteristics [54]. However, AM exhibits some significant disadvantages, including limited transparency and mechanical strength, poor standardization of preparation, risk for disease transmission, and biological variability (Table 7) [55]. There are extensive similarities between the basement membrane composition of AM and limbal niche, but AM lacks limbus-specific environmental factors, making it unsuitable as a surrogate niche for limbal stem cells [56]. In the studies on cell-based therapies of LSCD that have only been investigated in animal experiments, AM, with favorable results (Table 1, Table 3, and Table 4), has been used as a substrate for culture and transplantation of bone marrow-derived mesenchymal stem cells (MSCs) [34,35,38], epidermal stem cells (SCs) [46,47], immature dental pulp stem cells (IDPSCs) [49,50], and umbilical cord stem cells (UCSCs) [52].

**Table 6:** Potential biomaterials and carriers for ocular surface reconstruction.

Biological/Biosynthetic	Synthetic
Amniotic membrane [57]	Contact lenses [58]
Chemically cross-linked hyaluronic acid-based hydrogels [59]	Mebiol Gel (thermo-reversible polymer gel) [53]
Chitosan matrix/silver matrix/gold matrix [60]	Nanofiber scaffolds [40]
Collagen IV-coated plates [61]	Petrolatum gauze [24]
Collagen membranes [62]	Plastic [25]
Corneal stroma [63]	Poly(lactide-co-glycolide) electrospun scaffolds [64]
Fibrin [65]	Poly-ε-caprolactone electrospun scaffolds [66]
Human keratoplasty lenticules [67]	
Laminin-coated compressed collagen gel [68]	
Matrigel (reconstituted basement membrane extract) [69]	
Plastic compressed collagen [70]	
Recombinant human cross-linked collagen scaffold [71]	
Silk fibroin [72]	
Silk fibroin mixed with polyethylene glycol [72]	

The list of possibilities is not complete.

As a substitution for natural extracellular matrix, investigators have attempted to produce synthetic nanofiber scaffolds, primarily using electrospinning [66], with the purpose of supporting cellular growth in corneal engineering. Nanofibers are three-dimensional (3D) and exhibit an enormous surface area. Polycaprolactone, which is a degradable polyester, has been found to have sufficient mechanical strength, high biocompatibility, low production costs, and ease of use (Table 7) [73]. Polycaprolactone has proved to be a suitable substrate for culture of corneal [66], limbal [66], and conjuntival cells [35]. Zajiceva *et al*. cultured bone marrow-derived MSCs on 3D nanofiber scaffolds fabricated from polyamide and transplanted the sheets onto the cornea of LSCD mice models [40]. The viability and morphology of cells grown on these nanofibers were comparable with those grown on plastic. Recently, a protocol for the use of nanofiber scaffolds for the growth of MSCs and limbal stem cells, and for their transplantation onto a damaged ocular surface in a mouse model, has been described, demonstrating the potential for nanofibers in clinical studies [74]. There are no studies, however, that have used nanofiber scaffolds for ocular surface reconstruction in humans.

Fibrin, a degradable natural substrate, has been used as a culture membrane in the treatment of LSCD in humans [75,76]. Fibrin substrates provide several advantages, such as relatively high mechanical strength, a high degree of transparency, and rapid bioadsorbence (Table 7) [54]. Fibrin, compared to, for example, collagen, has been shown to promote growth, survival, and an undifferentiated phenotype of cultured LEC [77]. The value of this membrane in ocular surface reconstruction has been further supported in LSCD rabbit models, using bone marrow-derived MSCs [33] and epidermal SCs [45], and in mice with hair follicle-derived stem cells (HFDSCs) [48].

Most of the cell-based therapeutic strategies entail the use of underlying substrate scaffolds. However, carrier-free methods, without a supportive membrane, have also been applied. Polymers that are responsive to temperature can detach adherent cells by reducing the temperature from 37 °C to 20 °C [78]. Carrier-free techniques take advantage of adhesive properties of the basement membranes. It was demonstrated that the presence of β_1_ integrin in the carrier-free group is important for the attachment of cell sheets to the ocular surface [79]. Promising results with carrier-free transplantation in animal studies are reported using bone marrow-derived MSCs in rabbits [36] and embryonic SCs in pigs [43] and mice [41,42,44].

**Table 7:** Properties, advantages, and disadvantages of different carrier biomaterials and methods used in cell-based therapies of LSCD, explored in animal models.

Carriers/Methods	Transparency	Mechanical Strength	Elasticity	Advantages	Disadvantages
AM	+	++	+++	Stimulates cell growth, anti-inflammation, anti-angiogenesis, proper elasticity	Limited transparency, variable quality, risk of disease transmission, limited mechanical strength, poor standardization
Carrier-free method	N/A	N/A	N/A	Rapid adhesion, does not require preparation and standardization of membranes, does not require sutures	Possibility for detachment from the ocular surface in the early period after surgery
Fibrin gel	++	+++	+++	Proper transparency, good bioadsorbence, easy manipulation, good mechanical strength, elasticity, degradable	Possibility for immune response, risk for disease transmission
Nanofiber	++	++++	++	Good transparency, high mechanical strength, highly flexible, proper biocompatibility, easy to use, controlled shape and pore size, low cost	Limited elasticity, high cost

N/A indicates not applicable.

## 5. Cultured Bone Marrow-Derived Mesenchymal Stem Cells

Mesenchymal stem cells have multi-lineage potential [80]. Previous studies have reported that bone marrow-derived MSCs have a beneficial effect on the survival, growth, and proliferation of various types of cells, such as cardiac progenitor cells [81], neural stem cells [82], neurons [83], and Schwann cells [84]. Studies have demonstrated that *in vivo* administration of MSCs decreases the incidence of graft-*versus*-host disease in humans and mice [85,86], inhibits the manifestation of autoimmune diseases [87], impairs septic complications [88], and considerably counteracts rejection of allogeneic corneal allografts [89]. After *in vivo* application of MSCs, these cells migrate into the damaged area, thus supporting tissue healing [90].

The role of bone marrow-derived MSCs has also been investigated in corneal tissue regeneration. To date, as many as eight animal studies have been performed using this cell source for corneal repair following induced LSCD (Table 1). Various substrates and methods have been applied to transplant cultured MSC cells to damaged cornea of mice, rats, and rabbits, including AM [34,35,38], nanofiber scaffold [40], fibrin scaffold [33], carrier-free sheets [36], injection under transplanted AM [37], and intravenous injection through an ear vein [39].

Overall, the results obtained from animal experiments show that bone marrow-derived MSCs have a favorable effect with regard to cell differentiation into a corneal epithelial phenotype, improved corneal clarity, and reduced vascularization (Table 1). In one mouse study, with the short follow-up time of two weeks, the authors reported that transplantation of bone marrow-derived MSCs on nanofiber scaffold carriers supported the epithelial healing and inhibited local inflammatory reactions [40]. The other studies, with follow-up times ranging from one to six months, reported that the reconstruction of corneal epithelium after transplantation of bone-marrow derived MSCs was achieved in 90.6% (29/32) of the experimental rats [34,35,38] and 100% (31/31) of the experimental rabbits [33,36,37,39]. In rats with induced LSCD, where cultured cells were transplanted on AM, the improved corneal clarity was achieved in 87.5% (28/32) of the transplanted animals, and the cornea was completely transparent in 78.6% (22/28) of the animals [34,35,38]. However, no studies reported that the cornea was completely transparent after transplantation in rabbit LSCD models [33,36,37,39]. In one of these studies where MSCs were transplanted on a fibrin carrier, the iris was partially clear in 30% (3/10) and completely obscure in 70% (7/10) of the transplanted animals [33]. The studies in both rats and rabbits have also revealed that some neovascularization was observed in all transplanted eyes, with the best outcome being neovascularization limited to 2 mm central to the limbus 10 weeks after the transplantation [34].

It is speculated that the favorable effect of bone marrow-derived MSCs may be mediated by the intercellular signaling of epidermal growth factor (EGF) [91]. It has been suggested that EGF may be one of the most important mitogens of corneal epithelial cells [33,34]. Furthermore, bone marrow-derived MSCs induced to corneal lineage exhibited up-regulation of the putative limbal epithelial stem cell-specific genes p63 and β_1_-integrin, and protein levels of p63 and CK3 were increased [38]. Other investigators have reported similar findings with the up-regulation of key putative stem cell markers [33,34,36,37]. This may be particularly important in the light of the recent finding by Rama *et al*. that the phenotype of cultured LEC is critical to ensure successful reconstruction of the ocular surface following LSCD [76]. The authors found that cell cultures in which p63-bright cells constituted more than 3% of the total number of cells were associated with successful transplantation in 78% of patients. In contrast, cultures in which p63-bright cells made up 3% or less of the total number of cells, successful transplantation was only seen in 11% of patients. In conclusion, the investigations performed in animal experiments suggest that bone marrow-derived MSCs may serve as a possible stem cell source for corneal reconstruction in humans, however, neovascularization was a consistent feature following transplantation. 

## 6. Cultured Embryonic Stem Cells

Embryonic SCs are widely accepted as a significant cell source in tissue regeneration due to their great plasticity. A number of cell types have been induced from embryonic SCs *in vitro*, e.g., lung alveolar epithelial cells [92] and epithelial cells of the thymus [93]. It has also been demonstrated that embryonic SCs are capable of differentiating into corneal epithelial-like cells [94,95]. There are hitherto four studies that have investigated the potential of embryonic SCs for regeneration of the cornea in animal LSCD models (Table 2). In these studies, embryonic SCs were either cultured on collagen IV [41,42,43] or gelatin coated plates [44]. After culture, the carrier-free cell sheets were transplanted onto the corneas of mice [41,42,44] and pigs [43] (Table 2).

Following transplantation of cultured embryonic SCs onto corneas of LSCD animal models, re-epithelialization of the corneal surface with monolayer [41] and multilayer [42,43,44] epithelial-like cells was observed. The restored epithelium exhibited high levels of expression of CD44 and E-cadherin, which are important in corneal epithelial wound healing [41,42,44]. Furthermore, it has been demonstrated that embryonic SCs induced into epithelial-like cells expressed the basal limbal epithelial marker p63 [42,43] and the mature corneal epithelial marker CK12 [41,42,43,44].

Disadvantages of using embryonic SCs include difficulty of access, ethical concerns, high costs, immunogenicity, and risk of tumor formation [96]. None of the studies using embryonic SCs in animals have reported the degree of success in terms of number of animals with corneal reconstruction, or the effect on corneal transparency and neovascularization. Moreover, the follow-up time is very short (from one day to five weeks). Taken together, more studies with longer follow-up times, which also inform on the degree of success, are warranted prior to clinical trials.

## 7. Cultured Epidermal Stem Cells

Epidermal SCs have the remarkable ability to differentiate into other types of tissues [97]. Three studies have so far demonstrated the potential of epidermal SCs to regenerate the corneal surface following LSCD (Table 3). Two of the studies used AM for the culture and transplantation of epidermal SCs onto the cornea of goats [46,47], whereas the other used fibrin scaffold in rabbits [45]. These studies demonstrated that culture and transplantation of epidermal SCs onto damaged cornea successfully restored the corneal epithelium in 100% (22/22) of the animals. Moreover, the cornea became completely transparent with only mild neovascularization [45,46,47]. In one study, the corneal surface was intact with normal transparency for over three months [45]. In a study by Yang and colleagues, with a follow-up time to 30 months, the cornea was clear in three or four quadrants in 80% (8/10) of animals [46]. In a third study, with a follow-up time to 24 months, 71.4% (5/7) of the eyes of the treated animals had two or three quadrants of clear cornea [47]. Corneal perforation during the operation was reported in one animal [47]. No other complications were noted in any of the animals.

Following transplantation of the epidermal SCs onto the cornea of goats, the epidermal markers CK1/10 were down-regulated in the corneal stroma at 12 months, whereas the expression of the CK3, CK12, and PAX6 was up-regulated in the reconstructed epithelium [46]. The authors suggested that a possible mechanism of epidermal SCs in reconstruction of the damaged corneal epithelium involves the down-regulation of CK1/10 and up-regulation of PAX6. The PAX6 gene is involved in controlling eye formation during embryonic development [45,98,99], and recently the transduction of PAX6 in skin epithelial stem cells has been demonstrated to be adequate to transform epidermal SCs to limbal stem cell-like cells [45].

In conclusion, the results obtained with epidermal SCs in animal studies are very promising, with a high degree of success following transplantation in many animals, even with a follow-up period of 2.5 years [46,47]. Since epidermal SCs are also exceptionally easy to access, they may prove to be an excellent cell type for treating LSCD in humans.

## 8. Cultured Hair Follicle-Derived Stem Cells

The hair follicle harbors mesenchymal stem cells in the dermal papilla and connective tissue sheath that have large plasticity and can differentiate—given appropriate conditions *in vitro* and *in vivo*—into several cell lineages. These include chondrogenic, osteogenic, adipogenic, myogenic, neurogenic, and hematopoietic cell lineages [100,101,102]. In addition, the hair follicle comprises stem cells of epithelial origin, residing in the bulge region of the outer root sheath. The cells possess the ability to differentiate into hair follicles and sebaceous glands under physiological conditions. Following injury, however, these stem cells differentiated into epidermis [103,104,105].

By means of conditioned media harvested from corneal and limbal stromal fibroblasts, Meyer-Blazejewska *et al*. found that hair follicle-derived stem cells (HFSCs) were able to be reprogrammed *in vitro* into cells with a corneal epithelial phenotype [106]. In a follow-up study, the same research group performed *in vivo* experiments using a transgenic mouse model that allows HFSCs to change color upon differentiation to corneal epithelial cells, in which CK12 is expressed [48]. Hair follicle-derived stem cells were cultured on fibrin scaffolds and transplanted onto the cornea of mice with induced LSCD. The achieved results were promising, with cell differentiation into a corneal epithelial phenotype and suppression of vascularization and conjunctival ingrowth with reconstruction of the ocular surface in 87.5% (7/8) of the transplanted animals two weeks following transplantation.

Due to promising results in an animal study comprising as many as 31 mice and extremely easy access, HFSCs clearly warrant further investigations.

## 9. Cultured Immature Dental Pulp Stem Cells

Human immature dental pulp cells (IDPSCs) are capable of differentiation into a multitude of cell types, including neurons, smooth and skeletal muscle, cartilage, and bone [107]. There are two animal studies using human IDPSCs to treat LSCD in which the cells were cultured on AM and transplanted onto the damaged cornea of rabbits [49,50]. Human immature dental pulp cells expressed markers in common with LEC/corneal cells, such as ABCG2, β_1_-integrin, p63, and CK3/12 [50]. In 2009, Monteiro *et al*. [50] demonstrated that transplantation of IDPSCs resulted in reconstruction of the ocular surface in 100% (5/5) of experimental animals. The authors also reported gradual improvement in corneal transparency during a follow-up time of three months [50]. One year later, Gomes and colleagues showed that rabbit eyes after transplantation of IDPSCs exhibited well-organized corneal epithelium and improved corneal transparency in 100% (5/5) of animals with mild chemical burn damage, while control corneas developed total conjunctivalization and opacification [49]. In the animals with severe chemical burns, 75% (3/4) of transplanted eyes showed less organized and loose corneal epithelium and inflammatory cells within the superficial and stromal layers. Furthermore, one animal exhibited a thin corneal epithelium and superficial neovascularization [49].

Overall, these two studies using IDPSC have shown that the transplantation of tissue engineered IDPSC sheets could successfully restore the ocular surface in animal models of LSCD. Human IDPSC are relatively easy to access from the dental pulp; however, the need for extraction of the tooth is a clear disadvantage with this technology.

## 10. Cultured Umbilical Cord Stem Cells

There is only one study on the potential use of umbilical cord stem cells (UCSCs) to reverse LSCD in animals [52]. The UCSCs were cultured on AM and then transplanted onto the cornea of a LSCD rabbit model, resulting in regeneration of a clear corneal epithelium with a smooth surface and minimal corneal neovascularization in 66.7% (4/6) of the animals. Mild superficial inflammation was reported in one eye, whereas severe neovascularization was observed in the other. Furthermore, it was demonstrated that this new corneal smooth surface exhibited expression of normal corneal-specific markers CK3 and CK12, but not CK4 or CK1/10. Compared to embryonic SCs, umbilical cord stem cells have the advantage of being less immunogenic [108], non-tumorigenic [108], and ethically acceptable [52]. Compared to hair follicles and epidermal cells, the disadvantages of UCSCs include more complicated accessibility and allogeneic transplantation.

## 11. Cultured Orbital Fat-Derived Stem Cells

Multipotent stem cells have recently been successfully isolated and purified from human orbital fat tissue [109]. It has been demonstrated that the growth kinetics of orbital fat-derived stem cells (OFSCs) resemble those of bone marrow-derived MSCs, and that they share several surface markers [110]. Low immunogenicity of OFSC transplantation has been demonstrated in a xenotransplant model [110]. Furthermore, OFSCs possess adipogenic, chondrogenic, and osteogenic differentiation capacity, and are capable of differentiating into corneal epithelial cells *in vitro* [109]. So far, there is only one study on the potential use of OFSCs to treat damaged ocular surfaces in mice [51]. The authors reported that the topical administration and intra-limbal injection of OFSCs resulted in the reconstruction of clear corneal epithelium one week after treatment. It is suggested that inflammatory inhibition and corneal epithelial differentiation by OFSCs are responsible for corneal wound healing in the first few days, and that corneal stroma engraftment of OFSCs at a late stage is associated with corneal transparency [51]. The possibility of a topical approach to deliver OFSCs to reconstruct the ocular surface is particularly promising as it represents a non-invasive method. So far, few other non-invasive strategies have been suggested for the treatment of LSCD, and currently include the use of amniotic membrane extract [111], limbal fibroblast conditioned medium [112], and autologous serum [113], “a tonic for the ailing epithelium” [114].

## 12. Challenges and Future Perspectives

Over the past 10 years, a number of stem cell sources have been suggested for the treatment of ocular surface disorders. The clinical decision as to the optimal approach to treat LSCD has become challenging due to a precipitous increase in treatment options coupled with an almost absence of comparative studies. Comparisons between animal experiments of cell-based therapies of LSCD are difficult due to the following factors: (a) various methods for inducing LSCD in animals, (b) assorted culture techniques, (c) various transplantation methods, (d) differences in postoperative treatment, (e) disparities in follow-up time, and (f) huge differences in the presentation of experimental data. Increased standardization of these parameters will simplify the comparisons between animal experiments involving different stem cell sources, thereby encouraging corneal regenerative medicine.

Mechanisms through which cell-based therapies reconstruct the ocular surface are still elusive. The transplanted cells may substitute the progenitor/stem cells of the host for a period of time and/or revitalize the stem cells of the host, e.g., by secreting growth factors. There are several lines of evidence supporting the hypothesis that cultured cells transplanted onto the cornea primarily work by providing a favorable environment. The fact that LSCD can be successfully treated by a number of cell types implies that factors other than the choice of cell type may govern clinical success. The identification of factors secreted from cultured non-limbal epithelial cells that may be involved in the revitalization of limbal stem cells is an exciting future avenue for research.

It is likely that the phenotype of cultured non-limbal cells affects success following transplantation [76]. Studies on how various culture parameters affect the cell sheet, with particular emphasis on the phenotype, are warranted.

## 13. Conclusions

Animal experiments with epidermal SCs, HFSCs, IDPSCs, and bone marrow-derived MSCs have all shown promising results for the treatment of LSCD (Table 8). They represent an autologous source of cells in contrast to embryonic SCs and UCSCs. The long-term effects using embryonic SCs and UCSCs are unknown as none of the cell types have a follow-up time longer than five weeks. This contrasts sharply with the 2.5 year follow-up time for transplanted cultured epidermal SCs. Epidermal SCs and HFSCs both have the distinct benefit of exceptional ease of access. Coupled with promising results in many animals, these two types are particularly strong candidates for future clinical trials. Future research on these cells could include the development of a xenobiotic culture and storage [115,116,117,118,119,120] system that can keep the cells in a relatively undifferentiated state [76], while maintaining sufficient strength to be suitable for transplantation. Such a system would increase the safety [121], flexibility [122], global impact [123], and, most likely, the clinical results of the transplants [76].

**Table 8:** Overall success in ocular surface reconstruction using different stem cell sources.

Types of Stem Cells	Success	Complications (Number of Animals)	Ease of Access	Number of Animals (Number of Studies)	Autologous Source	Ethical Concerns
Bone Marrow-Derived MSCs	+++	–	++	63 (8) ^1^	Yes	No
Embryonic SCs	+	Mild immune reaction *	+	25 (4) ^2^	No	Yes
Epidermal SCs	++++	Perforation (1)	++++	22 (3)	Yes	No
HFSCs	+++	–	++++	31 (1)	Yes	No
IDPSCs	+++	–	++	14 (2)	Yes	No
OFSCs	++	–	++	12 (1)	Yes	No
UCSCs	++	Mild superficial inflammation (1)	++	6 (1)	No	No

^1^ number of animals not reported in two studies; ^2^ number of animals not reported in one study; * number of animals not reported; HFSCs, hair follicle-derived stem cells; MSCs, mesenchymal stem cells; SCs, stem cells; OFSCs, orbital fat-derived stem cells; UCSCs, umbilical cord stem cells; +: low degree; ++: moderate degree; +++: high degree; ++++: very high degree.
